# Exploring Metalinguistic Awareness in School-Aged Autistic Children: Insights from Grammatical Judgment

**DOI:** 10.1007/s10803-024-06569-y

**Published:** 2024-10-07

**Authors:** Pauline Wolfer, Franziska Baumeister, Nicola Rudelli, Grace Corrigan, Letitia R. Naigles, Stephanie Durrleman

**Affiliations:** 1 Autism, Bilingualism, Cognitive and Communicative Development Lab (ABCCD), Faculty of Science and Medicine, University of Fribourg, Fribourg, Switzerland; 2 Department of Education and Learning/University of Teacher Education, Competence Centre for School, Social and Educational Needs (BESS), University of Applied Sciences and Arts of Southern Switzerland (SUPSI), Locarno, Switzerland; 3 Psychological Sciences, University of Connecticut, Storrs, CT USA

**Keywords:** Autism, Metalinguistic awareness, Grammatical judgment task, Morphosyntactic awareness, Semantic awareness

## Abstract

Metalinguistic awareness, the ability to manipulate and reflect upon language, remains largely unexplored in the autistic population. To address this gap, this observational cross-sectional study examines the metalinguistic abilities of school-aged autistic children in comparison to neurotypical peers in a novel tablet-based Grammatical Judgment Task (GJT) of reduced linguistic complexity engaging two kinds of metacognitive resources. Children had to judge non-verbally whether pre-recorded sentences were grammatically correct or not, following the traditional GJT paradigm assessing *metamorphosyntactic* skills. In addition, sentences with anomalous meaning that were either grammatically correct or grammatically incorrect were introduced to test *metasemantic* knowledge. Findings reveal no difference in performance between the groups, with participants performing on average above chance level both on the sentences assessing mere metamorphosyntactic skills and on the sentences placing an additional demand on metasemantics. This study shows that autistic individuals are able to mobilize metalinguistic resources when tested via a task of reduced linguistic complexity.

**Supplementary Information:**

The online version contains supplementary material available at 10.1007/s10803-024-06569-y.

Autistic individuals experience challenges in communication and social interactions (American Psychiatric Association, 2013), including difficulties using language in context (i.e., pragmatics) (Bartak et al., 1975). Much work striving to establish communicative profiles in individuals with Autism Spectrum Disorder (ASD)^1^ has focused on these pragmatic impairments, but less is known about how they understand, evaluate, and manipulate language beyond social contexts (i.e., metalinguistic awareness), another important communicative skill which is the focus of the current study. To begin, we will describe metalinguistic awareness, highlight its importance for communication and language, and explain why it is relevant to further investigate this ability in school-aged autistic children. Then, we will undertake an experimental study of children with ASD, who reportedly struggle more with meaning than form (Naigles & Tek, 2017), and thus we expect to show relatively strong *metamorphosyntactic* abilities as compared to their *metasemantic* skills.

Metalinguistic awareness refers to the ability to reflect on language as an object of thought (Tunmer & Herriman, 1984). This awareness implies distancing oneself from language (Ramirez et al., 2014), so as to be able to evaluate and manipulate linguistic elements at different levels: for instance, *morphosyntactic awareness* is activated when detecting and correcting grammatical mistakes (Gombert, 1990); *semantic awareness* is involved when one is able to detach word form from meaning, subsequently enabling the use and comprehension of figurative language. Whether consciously or automatically activated in daily-life interactions, in laboratory-based settings, or in educational environments, metalinguistic awareness involves a range of skills that enable the examination of language structure and use beyond the realms of language production and comprehension (Sinclair, 1986).

The impact of these higher-order skills is not limited to language in and of itself – be it written or oral – but extends to communication in a broader sense. In view of its importance for effective communication, the study of metalinguistic awareness in populations experiencing communication challenges, such as individuals on the Autism Spectrum, seems particularly relevant. Indeed, these abilities play a crucial role not only in understanding the linguistic message within a communicative context but also in grasping the nuances of the interactional context, as well as the hidden intention(s), to formulate an adequate response. Metalinguistic awareness is constantly mobilized to repair miscommunications, adapt to the conversation and to the communicative partners (Patterson, 2011). For the speaker, notably, being able to detect grammatical mistakes in a sentence is the first step for self-correction, which will foster message intelligibility and discourse coherence maintenance for the interlocutor. Being able to spot grammatical mistakes such as morphological omissions or word reversal, can also inform and advise a speaker about the language proficiency of their communicative partner (e.g., presence of a language impairment, use of another language variety, partner’s register preference). Just like body language interpretation and facial emotion processing, this enables capturing all the cues of a communicative situation and allows for smooth adaptation essential for an effective communication. The ramifications of metalinguistic awareness extend beyond that of communication *per se*, being also an important predictor of academic achievement (Al-Ahdal & Almarshedi, 2022), vocabulary learning (Altman et al., 2018; Ramirez et al., 2014), spelling performance (McNeill & Everatt, 2013), and reading comprehension (Dong et al., 2020).

In an attempt to better characterize this construct (Bialystok, 2001; Sinclair, 1986), Bialystok & Ryan (1985) identified two crucial dimensions involved in metalinguistic activities within a cognitive framework: *(linguistic) knowledge analysis*, referring to the knowledge of the formal aspects of the language, and *cognitive control (of linguistic processes)*, corresponding to the ability to pay attention to and to monitor these specific linguistic representations, including in ambiguous or misleading contexts (Bialystok, 1986; Bialystok & Ryan, 1985). These resources are deemed to be present in every metalinguistic task, albeit to various degrees: in the case of a morphological awareness task for instance, Friesen and Bialystok (2012) explain that increasing demands in *analyzed knowledge* can be gradually manipulated by varying the degree of complexity of the linguistic information to be judged, or in asking an individual to locate the (potential) grammatical mistake, correct it or/and justify their answer verbally, rather than “just” asking for a judgment. Different levels of *control* can be set by manipulating the task’s cognitive complexity, such as adding salient distracting information that needs to be ignored to perform adequately on the task (Friesen & Bialystok, 2012). While the authors themselves retrospectively qualify the terms *analysis* and *control* as “poor choices” (Bialystok, 2024, p. 1), these terms nevertheless reflect the intertwinement and the importance of both the cognitive and linguistic components at play: Metalinguistic tasks are neither purely linguistic nor purely cognitive, as in grammatical judgment tasks for instance, both representations of the linguistic structures and tuning attention to these representations are arguably required to perform successfully (Bialystok, 2024).

Assuming that these abilities are indeed inherent to any task testing a form of metalinguistic awareness, it is evident that a certain level of linguistic and/or cognitive development may be required to perform successfully (Cummins, 1978; Edwards & Kirkpatrick, 1999). Indeed, these higher-order linguistic skills mature over time, emerging during the later phases of language development and build upon prior linguistic knowledge (Duncan et al., 2009). While some studies identify early signs of metalinguistic awareness in children as young as three years old (Patterson, 2011; Sinclair, 1986), the majority of studies suggest that these skills begin to emerge when children enter primary school at around five to six years old, and continue to develop gradually throughout childhood (Melogno et al., 2022) and even beyond (Edwards & Kirkpatrick, 1999). A pivotal milestone is identified around the age of seven, indicating an important improvement in metacompetence, likely linked to broader general cognitive development (Cummins, 2014; Roehr-Brackin, 2024).

Despite its relevance for successful communication and its ubiquity in everyday life, metalinguistic awareness has been rarely explored in neurodivergent populations, such as Autism Spectrum Disorder, for whom communicative challenges have been specifically identified as a diagnostic criterion in the DSM-5 (American Psychiatric Association, 2013). There have been numerous attempts to better characterize the linguistic profiles of children on the spectrum (Tager-Flusberg, 2006; Tager-Flusberg & Joseph, 2003), which have led to accurate depictions of the heterogenous phonological, lexical, morphosyntactic and pragmatic abilities of autistic individuals (Schaeffer et al., 2023; Silleresi, 2023; Sukenik & Tuller, 2023); however, there is still a gap in our understanding about whether and how autistic individuals evaluate, manipulate, and reflect upon language at these different levels. Indeed, unlike children with Typical Development (TD) who may develop these later-emerging metalinguistic skills through typical language acquisition processes in interraction with cognitive and educational factors (Melogno et al., 2022), children with ASD often experience atypical language and cognitive development trajectories (Kissine et al., 2023; Tager-Flusberg, 1999), which could potentially impact the growth and integrity of metalinguistic knowledge.

A preliminary study assessing metalinguistic ability in general revealed that twenty autistic children aged nine to seventeen years old performed significantly below their 18 aged-matched neurotypical peers of similar cognitive abilities, in a range of standardized tasks involving complex linguistic skills at the morphosyntactic and semantic levels, such as inferential language understanding and ambiguity resolution tasks (Lewis et al., 2007). While this was taken to suggest that autistic individuals may face difficulties in the realm of metalinguistic abilities, the study did not include any direct assessment of the participants’ structural linguistic skills, such as their current receptive vocabulary breadth or receptive morphosyntactic skills. As such, this omission leaves open the possibility that these skills could have influenced the participants’ metalinguistic performance, given the reported decisive role of structural linguistic skills in metalinguistic awareness tasks, particularly at the semantic level (Kalandadze et al., 2018), or at the lexical and morphosyntactic levels (Smith & Tager-Flusberg, 1982). Additionally, it is worth noting that half of the tasks in the study by Lewis and colleagues required a verbal response, a modality that could penalize children with language impairment, who are estimated to represent more than two-thirds of the autistic population (Rapin & Dunn, 2003).

In the limited literature on metalinguistic awareness in autistic individuals, *metasemantic* awareness might be the most extensively documented, driven by a substantial body of literature exploring how children with ASD understand figurative language (e.g., sentences with non-literal meaning, irony, metaphors, idioms), where the intended meaning differs from the litteral utterance (e.g., exclaiming “What a lovely day!” when it is raining). Recent reviews and meta-analyses consistently highlight deficits in mastering these complex higher-order skills among autistic individuals (Kalandadze et al., 2018; Melogno et al., 2022; Morsanyi et al., 2020) in favor of a literalist bias (i.e., tendency to interpret figurative language in a literal way) (Vicente & Martín-González, 2021). Yet, Lampri and colleagues (2023) urge researchers to design and use low-verbal tasks (i.e., with minimal linguistic demands) to accurately capture the specific abilities targeted by the assessment, rather than “their verbal competence *per se*” (p. 12), a strategy applied in psycholinguistic research to reduce the linguistic confound in assessing various cognitive skills such as non-verbal reasoning (Silleresi, 2023), Theory of Mind (Burnel et al., 2018), and executive functions (Kaushanskaya et al., 2017; MacDonald & Christiansen, 2002). The need for “purer” assessments, i.e., devoid of potential confounding factors, has been emphasized in the context of metalinguistic awareness as well: Singson and colleagues (2000), for instance, suggest the use of a Grammatical Judgment Task (GJT) (i.e., during which participants are required to decide whether a heard sentence is grammatically correct or not), instead of a sentence completion task to assess morphosyntactic awareness in school-aged neurotypical children, thereby avoiding any additional burden on verbal short-term memory (Singson et al., 2000). The careful design of a GJT with a non-verbal response from the participant, offers distinct advantages as it enables the assessment of linguistic and metalinguistic competencies with minimal reliance on language resources. Indeed, previous work has underscored the dual nature of these judgment tasks, which involve both mere structural language abilities and metacognitive processing skills (Schachter & Yip, 1990): GJTs require a conscious examination of the linguistic structure via a heightened attention towards the linguistic content, and its comparison to the linguistic representations held in memory, in order to make an explicit accurate judgment. These analytical abilities are deemed to belong to a more general non-linguistic cognitive system (Bever, 1970) and to engage extragrammatical factors (Schachter & Yip, 1990; Tremblay, 2005); thus, they might reflect metacompetence beyond solely linguistic knowledge.

However, despite their potential to inform on metalinguistic abilities, these GJTs have seldom been used in the autistic population, except for two studies focusing on older children aged eleven to thirteen (Ambridge et al., 2015) and adolescents aged ten to sixteen (Eigsti & Bennetto, 2009). When employed, the GJT was primarily used as a tool to specifically assess structural language abilities, aiming to identify whether and which specific morphosyntactic structures might be affected in children with ASD. For instance, Eigsti and Bennetto (2009) used a GJT to more precisely pinpoint subtle grammatical structures with which children and adolescents with ASD may struggle (e.g., present progressive markings). Building on this work, Ambridge and colleagues (2015) reached the same conclusion by showing that children with ASD aged 11 to 13 had difficulties detecting specific grammatical errors (e.g. verb argument structure overgeneralization errors, e.g., *Lisa fell the cup off the shelf) which could not be attributed to more global cognitive delays or even to a potential phonological impairments (i.e., their poorer performance does not stem from being unable to detect grammatical morphemes). Interestingly, the authors acknowledged that due to the dual nature of the task in solliciting both linguistic and metalinguistic abilities, it is possible that children with ASD “might be impaired not on grammar *per se*, but on the particular paradigm used to assess it in this study” (p. 12), namely a GJT.

The current study seeks to gain a more comprehensive understanding of metalinguistic abilities in children with ASD by taking advantage of Bialystok and Ryan’s cognitive framework (1985) and experimental GJT design in other work on bilingual populations (Bialystok, 1986; Bialystok et al., 2014; Hermanto et al., 2012) to decipher the respective performance of autistic and neurotypical children in their *metamorphosyntactic* and *metasemantic* skills in a single GJT. To this aim, a novel GJT which limits the impact of grammar *per se* was created, reducing the lexical and morphosyntactic processing demands inherent to the task (i.e., use of short sentences with simple-clause syntactic structure and simple vocabulary notably) as well as reducing verbal short-term memory load (by adhering to short sentences). Proposing such a GJT with a non-verbal response paradigm further avoids penalizing children with ASD for their expressive and socio-communicative impairments. In addition, the task was developed on a tablet, so as to take into consideration the preference of digital technologies exhibited by the autistic population (Scholle et al., 2020). To gain more knowledge on the participants’ *metasemantic* abilities at a surface level and its interaction with *metamorphosyntactic* abilities, sentences with anomalous meaning of either correct, or incorrect grammatical structure, were included. These sentences require participants to make grammatical judgments while ignoring their meaning, a *metasemantic* skill mobilized in the very first steps of pragmatic inferencing: in the case of metaphor understanding for instance, the processing of the literal meaning should be repressed, to subsequently (a) analyse the linguistic elements separately and (b) reconcile the literal anomaly with the intended message. This GJT therefore engages both *metamorphosyntactic* and *metasemantic* skills, because it requires the participant to make the distinction between the morphosyntactic and semantic dimensions of the sentence heard to perform accurately.

Thus, this work explores the metalinguistic skills of 6 to 12 years old autistic children, in comparison to age-matched neurotypical peers and taking their socioeconomic status proxy, non-verbal reasoning and overall receptive morphosyntactic skills into account, using a novel digitalized GJT with reduced linguistic complexity and limited burden in verbal short-term memory. Specifically, this observational cross-sectional study with a case-control design will investigate (1) whether the groups differed in the general performance on the task, and (2) whether this performance was differently impacted by the type of sentence. We hypothesized that children with ASD would perform on par with their non-autistic peers on the traditional part of the task (i.e., they would adequately judge the items that are semantically appropriate but that can contain a grammatical mistake), as the linguistic difficulty of the items has been reduced. However, we predict that autistic children may exhibit poorer performance than the neurotypical children on the part of the task engaging additional *metasemantic* resources (i.e., sentences with misleading odd meaning), given the reported difficulties in processing sentences with odd meaning (Kalandadze et al., 2018; Lewis et al., 2007; Morsanyi et al., 2020).

## Methods

### Participants

#### Sample

Thirty-eight autistic children aged 6;2–11;11 (Mean = 9;4; SD = 1;8) and ninety neurotypical children aged 6;0–11;7 (Mean = 8;7 SD = 1;8) took part in the study. The neurotypical children did not have any suspicion or diagnosis of Autism Spectrum Disorder or any other neurodevelopmental disorder. All participants had reported no uncorrected vision and hearing. Prior to the study, all autistic participants had received a diagnosis of ASD, established by a professional clinician (e.g., psychiatrist, psychologist). 68% (*N* = 26) of the autistic individuals had been diagnosed with a standardized tool known to the caregivers (*Autism Diagnostic Observation Schedule (ADOS-2)* (Lord et al., 2012): *N* = 21, *Autism Diagnostic Interview Revised ADI-R* (Lord et al., 1994): *N* = 3, *Vineland Adaptive Behavior Scale (VABS)* (McKinlay, 2011): *N* = 1, *Parents’ Evaluation of Developmental Status (PEDS)* (Glascoe, 1997): *N* = 1). For the remaining 32% of autistic participants, parents were able to provide an official report stating the Autism diagnosis and/or participants had scored equal to or above the threshold of 15 on the *Social Communication Questionnaire (SCQ)* (Rutter et al., 2003), an adequate cut-off to confirm a diagnosis in 4–18 years old children (Allen et al., 2007). There was an imbalance in the sex ratio between groups, with more male participants in the autistic group.

#### Socioeconomic Status

Parental educational level was used as an index for socioeconomic status. This is considered a reliable indicator due to its strong correlation with family income (Hauser & Warren, 1997) and association with children’s academic achievement (Sirin, 2005), brain function and cognitive ability (Cermakova et al., 2023). Parents were asked to select the highest degree of education they had completed, which was then transformed into a value on a 5-point Likert scale: (1) elementary school, (2) middle school, (3) high school, (4) post-secondary degree, (5) university. The higher value between the two caregivers was used for analysis.

#### Origin of the Sample

As part of an international multi-site project, testing took place in Switzerland, Germany, France, the UK and the US. The participants were tested in either English (9.4%), French (31.2%), German (49.2%), or Italian (10.2%). Given the consistent multilingual effects on tasks involving meta(-morphosyntactic) knowledge (Adesope et al., 2010; Bialystok, 2001), we recorded the participants’ linguistic background using the Q-BEx (De Cat et al., 2022), a parental questionnaire enabling a thorough and detailed characterization of the participants’ linguistic experiences. Around one-third of each group (36%) was exposed to a second language for more than 20% of their lifetime, a cut-off that has been used in research to define the bilingual group (Hantman et al., 2023). Additional languages children were exposed to are presented in Online Appendix A, detailed descriptive statistics of the sample are reported in Table 1.

**Table 1:** Descriptive statistics of the sample, cognitive and linguistic measures

	Autistic children (N = 38)	Non-Autistic children (N = 90)
**Age (months)**		
Mean ± SD	112 ± 20.4	103 ± 20.1
Median [Min, Max]	112 [74.0, 143]	102 [72.0, 139]
**Sex assigned at birth**		
F	3 (7.9%)	46 (51.1%)
M	35 (92.1%)	44 (48.9%)
**Parental educational level (min 1 - max 5)**		
Mean ± SD	4.21 ± 1.09	4.57 ± 0.90
Median [Min, Max]	5 [1, 5]	5 [1, 5]
**Language of administration**		
English	5 (13.2%)	7 (7.8%)
French	17 (44.7%)	23 (25.6%)
German	14 (36.8%)	49 (54.4%)
Italian	2 (5.3%)	11 (12.2%)
**Linguistic background**		
Monolinguals (Exposure to a 2nd language < 20% lifetime)	24 (64%)	58 (64%)
Multilinguals (Exposure to a 2nd language ≥ 20% lifetime)	14 (36%)	32 (36%)
**Receptive morphosyntax (TROG**,** z-score)**		
Mean ± SD	-0.44 ± 1.37	0.12 ± 1.32
Median [Min, Max]	-0.30 [-3.00, 1.60]	0.20 [-3.00, 2.80]
**Non-verbal IQ (Raven’s 2 score)**		
Mean ± SD	98.3 ± 15.5	99.7 ± 12.2
Median [Min, Max]	96.5 [77, 133]	102 [71, 122]

### Procedure and Measures

The tasks were part of a larger protocolaiming to test the linguistic and cognitive development of children with ASD. Each participant was individually tested in a quiet room, always accompanied by an experimenter to guide them through the tasks. All tests were administered in one of four language versions (English, French, German and Italian), to enable testing in different regions, and all children were tested in their most proficient language, which they all acquired before the age of three. In addition to the newly created *Grammatical Judgment Task*, participants non-verbal reasoning and receptive morphosyntactic skills were tested.

#### Grammatical Judgment Task (GJT)

To test metalinguistic abilities at the morphosyntactic and semantic levels, a new GJT was created, inspired by Atchley and colleagues (2006) and later by Bialystok and colleagues’ “sentence-judgment task” (Atchley et al., 2006; Bialystok et al., 2014). The GJT was embedded in a so-called serious game designed for tablet use. Tablet assessment was preferred because (1) it fosters *systematic and reproducible testing* by using of pre-recorded instructions, items, and feedback, and enabled control of the quantity and quality of instructions provided to each participant and across language versions; (2) it improves *accuracy* (Germine et al., 2019) by eliminating human error during scoring, as the process was automatized; (3) it allowed the experimenter to act as a partner of the session rather than a “judge” of the participant’s abilities, thus circumventing social challenges and additional distress face-to-face interaction impose on autistic individuals (Pinchevski & Peters, 2016); (4) tablet-based interventions and assessments have been proven effective in children with ASD (Alzrayer et al., 2014), who tend to be particularly drawn to digital technologies (Scholle et al., 2020). Participants were presented with a virtual classroom where a human-like 3D female teacher character with natural human voice introduced the task. A friendly little monster appeared on the screen, and participants were told that the monster was a new pupil trying to learn their language, and that they had to help the monster to improve. Participants had to listen to a pre-recorded sentence enunciated by the monster and to select either a picture of a sweet to feed them (i.e., if the sentence was grammatically correct) or a sock (i.e., if it was grammatically incorrect). It was specified that the monster disliked socks but that it should be corrected when making mistakes so that it could improve. To simplify the instructions and avoid biasing or confusing younger children unfamiliar with the concept, we decided to not use the term “grammatically correct” or “grammar” throughout the task’s explanations. Experimenters were allowed to repeat or rephrase the instructions, as close as the given instructions, and to answer the participants’ clarification questions. The task started with a familiarization phase of three to six practice items (depending on the participants’ performance) with corrective and explanatory feedback, to ensure that the participants understood that the judgment had to be made based on the grammar of the heard sentence. Thirty-two test items arranged in random order were then presented.

#### GJT Stimuli Specifications

Half of the 32 trials constituted a traditional GJT, with sentences being either grammatically correct (*N* = 8) or incorrect (*N* = 8), but all semantically appropriate (*N* = 16). The grammatical errors could be either of a syntactic nature (i.e., determiner-noun reversal, e.g., “she is picking flowers in *garden the*”), or of a morphological kind (i.e., subject-verb agreement, e.g., “the children buy*s*”). These two errors were selected as they were easily reproducible in all four languages in which the task was created. Moreover, as syntactic errors are easier to detect than morphological ones, by including errors of both types we were able to devise a task that was of alternating levels of difficulty. The other half of the sentences (*N* = 16) were either grammatically correct (*N* = 8) or incorrect (*N* = 8), but semantically anomalous. As in the study by Bialystok et al. (2014), semantic anomaly was created by an impossible or unrealistic pairing of the action with the agent (e.g., “the *shoe* is *cooking*”). Moreover, a considerable effort was made to reduce the linguistic complexity of the task, to avoid penalizing children with linguistic impairments, and to tap into metacompetence rather than linguistic abilities. The sentences were kept short (i.e., between nine to twelve syllables) to limit length effects, which have been reported to have a greater impact on performance in the autistic population (Eigsti & Bennetto, 2009). The sentences were also restricted to a simple clause syntactic structure and contained only verbs in the present tense, as autistic children have been shown to experience difficulties with past tense (Durrleman & Zufferey, 2009; Roberts et al., 2004). Finally, the lexical items were selected from the Mac-Arthur Bates Checklist Inventory MB-CDI (Fenson et al., 1994) and its respective adaptations in all targeted languages i.e., French (Kern et al., 2010), German (Szagun et al., 2014), Italian (Rinaldi et al., 2019), in order to reduce the lexical difficulty to words emerging in the first years of life. The instructions and stimuli were recorded for each language by a native speaker in a sound-proof room, then edited with Audacity software (Audacity Team, 2014) for normalization and voice pitch increase to create a slightly robotic, higher-pitched yet natural voice corresponding to a monster’s voice. An example of audio stimulus in each language is available under https://osf.io/wnsdc/?view_only=4baca7db03d24759b4d695b59990c112.

#### Language Versions

An initial 40-items GJT was created in French (i.e., 10 sentences per sentence type), which was then consequently adapted into English, German, and Italian with the same structure and characteristics to yield comparable GJTs across the four languages. Some lexical items (nouns and verbs) varied between languages, however, to fulfill the criterion of a maximum 12 syllables per sentence. Prior to the study, ten healthy native speakers of the four languages and aged 18–35, with no known neurological history, piloted the task and performed at ceiling, as expected. Consequently, two items per sentence type were removed, resulting in the final 32-item GJT now available in the four languages of interest. All instructions were translated from French, ensuring that the same amount of information was provided to all participants in each language. An example of stimuli is presented in Table 2, and a complete item list in all languages is available in Online Appendix B. One item of the English version was removed from all analyses due to an error subsequently identified in the wording^2^.

**Table 2:** Examples of the four sentence types of the 32-item GJT task, made equivalent in French, English, German, and Italian. There were 8 items per sentence type. Sentences appeared in a random order

	(a) grammatically correct, semantically appropriate	(b) grammatically incorrect, semantically appropriate	(c) grammatically correct, semantically odd	(d) grammatically incorrect, semantically odd
	G + S+	G- S+	G + S-	G- S-
English	Mark goes to the swimming pool with a friend.	Tonight there are clouds and stars in sky *the*.	The *broom* wakes up every morning.	The rain *climb* tonight in the sandbox.
French	Marc va à la piscine avec un ami.	La nuit il y a nuages *des* dans le ciel.	Le *balai* se réveille tous les matins.	La pluie *grimpons* ce soir dans le caillou.
German	Mark geht mit einem Freund ins Schwimmbad.	Es gibt in Nacht *der* Wolken am Himmel.	Der *Besen* wacht jeden Morgen auf.	Der Regen *klettern* heute Nacht in den Stein.
Italian	Marco va al mare con un amico.	Di notte sono stelle *ci* in cielo.	La *scopa* si sveglia la mattina.	La pioggia si *arrampicare* sul sasso

#### GJT Scoring

Scoring was automatically collected by the tablet device and stored in a csv file. For each item individually, a score of “1” was allotted to a correct response (i.e., the participant accurately selected the *candy* when the sentence was grammatically correct or the *sock* picture when it was grammatically incorrect, irrespective of the meaning) and “0” for an incorrect response. Only participants who completed all 32 items were considered in the analyses (*N* = 38 children with ASD, *N* = 90 children with TD). The overall performance for a participant therefore corresponded to the sum of correct responses (i.e., where the value for an item worth “1”) on all items, yielding a maximum score of 32 (8 items per sentence type). As judgment had to be made on the grammatical correctness only, there were equal opportunities to select the candy (50% of the trials) and the sock (50%).

#### Morphosyntactic Abilities Assessment

The participants were administered the *Test For Reception of Grammar (TROG)* version 2 (Bishop, 2003) and its respective adaptations in French (Lecocq, 1996), German (Fox-Boyer, 2006), and Italian (Suraniti et al., 2009), to assess their general receptive morphosyntactic skills in the language of test. The children listened to pre-recorded sentences of gradually increasing complexity involving various syntactic structures (e.g., negative sentences, “X but Y”, “neither nor”, relative clause in the subject and in the object, “not only X but also Y”, pronoun gender/number, etc.) and were asked to select the appropriate image from four choices displayed on screen. None of the items of the TROG assessed word order reversal nor subject-verb agreement. Given the variations in scoring systems, target structures, and total item numbers across the different language versions, Z-scores were used to allow for comparisons between participants of all languages.

#### Non-Verbal Reasoning Assessment

A non-verbal IQ score (Mean = 100, SD ± 15) reflecting fluid reasoning was obtained through the short version of the digitalized *Raven’s Progressive Matrices Second Edition* (Raven’s 2) (Raven et al., 2018). This reliable, relatively short task of graduated complexity requires the participant to select, amongst five choices, the piece of the puzzle that best fits the pattern of a big picture displayed on the screen in which one piece was missing. It has been shown to accurately reflect the non-verbal reasoning skills of autistic individuals across different intellectual profiles (*see* Silleresi, 2023*for an informative discussion on why this tool is particularly appropriate when testing children with ASD*). The performance of each group on the linguistic and non-verbal measures are presented in Table 1.

### Statistical Analyses

To assess whether groups differed in their overall performance, and whether their accuracy was differently impacted by the sentence types, a binomial generalized mixed-effects model with a logit function was implemented and was estimated using LM and BOBYQA optimizer. All statistical analyses were performed in R using R Studio (Version 2023.12.0 + 369) with the lme4 package (Bates et al., 2015).

#### Model Specifications

Group (children with ASD/children with TD), grammatical correctness (grammatically correct versus incorrect) and semantic appropriateness (semantically appropriate versus semantically odd) were entered as fixed effects, together with two- and three- way interaction between these three factors. The goal was to investigate (1) whether the groups differed in their overall performance, (2) whether the fact that a sentence is either grammatically correct or incorrect *(i.e.*,* grammaticality effect)* or semantically appropriate or inappropriate *(i.e.*,* effect of semantic appropriateness)* impacted the performance, and (3) whether these effects of grammaticality and semantic appropriateness differed between the groups. To ensure that the results were not driven by differences between groups in key variables, the following factors were entered as covariates: biological age (in months), non-verbal IQ (Ravens’ 2 score), general receptive morphosyntactic skills (TROG z-score), socioeconomic status proxy (Likert scale score 1–5), language of administration (English, French, German or Italian) as well as bilingual status (monolingual/bilingual). The model also had by participant and by item random intercepts, a by item random slope for the effect of group and by participant random slopes for the effects of grammaticality and semantic appropriateness to account for their variability beyond the fixed effects. Additional information on the model selection, contrast coding, and model checks is available in Online Appendix C. Complete model formulas are available under https://osf.io/wnsdc/?view_only=4baca7db03d24759b4d695b59990c112.

## Results

**Fig. 1:**
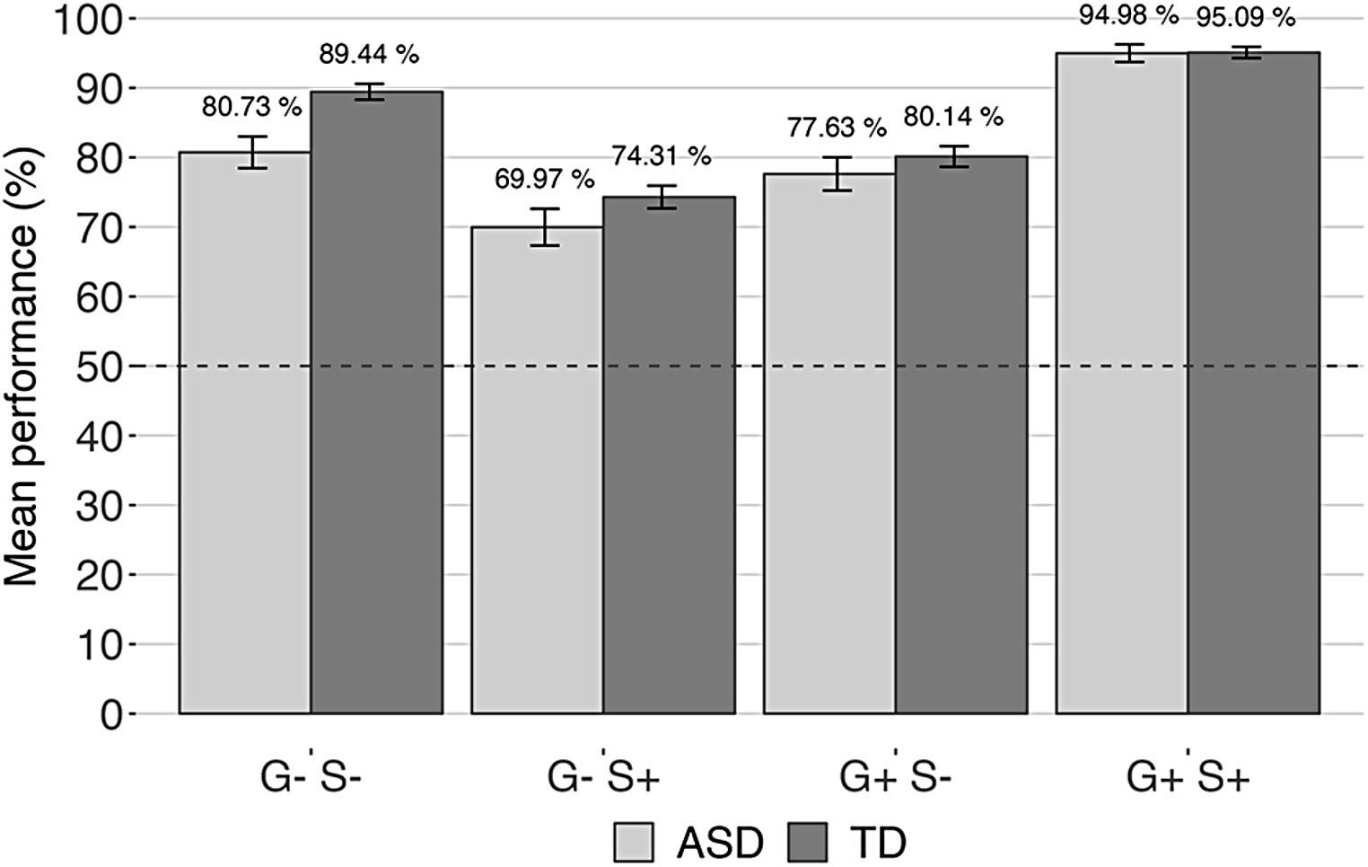
Performance (i.e., mean of correct responses) per groups and sentence types. G+/G- is used for grammatically correct/incorrect sentences, S+/S- for semantically appropriate/odd sentences. Exact percentages of correct responses are displayed above each bar

The two groups seemed to perform above chance level on all sentence types. The group of children with ASD produced on average 19.22% of incorrect responses, while the mean proportion of incorrect response was of 15.24% for the group of children with TD (Fig. 1 – see Online Appendix D for the distribution of performance by group and sentence type).

The model did not show any significant effect of group (β= - 0.36, SE = 0.30, *p* > .05) when accounting for socioeconomic status, non-verbal IQ, receptive morphosyntactic skills and the language of test (Table 3). However, age (β = 1.06, SE = 0.14, *p* < .001) and non-verbal reasoning (β = 0.58, SE = 0.15, *p* < .001) were significant positive predictors of the performance. Receptive morphosyntactic skills approached significance level (β = 0.21, SE = 0.11, *p* = .05).

**Table 3:** Fixed effects of the GLMM output

	Performance					
	Odds Ratios	CI	β	Std. Err	Z value	*p*
(Intercept)	15.97	10.13–25.16	2.77	0.23	11.94	**< 0.001**
Group	0.70	0.39–1.26	-0.36	0.30	-1.19	0.24
**Gramm_correctness**	**0.32**	**0.18–0.59**	**-1.14**	**0.31**	**-3.68**	**< 0.001**
**Sem_appropriateness**	**0.61**	**0.38–0.96**	**-0.50**	**0.23**	**-2.15**	**0.03**
Group*gramm	0.47	0.17–1.25	-0.76	0.50	-1.51	0.13
Group*sem	0.57	0.31–1.05	-0.56	0.31	-1.81	0.07
Gramm*sem	**29.49**	**10.72–81.11**	**3.38**	**0.52**	**6.56**	**< 0.001**
**Age**	**2.88**	**2.20–3.77**	**1.96**	**0.14**	**7.74**	**< 0.001**
SES	1.30	0.99–1.70	0.26	0.11	1.90	0.06
**Non-verbal IQ**	**1.78**	**1.33–2.37**	**0.58**	**1.31**	**3.92**	**< 0.001**
Rec. morphosyntax	1.22	0.99–1.52	0.20	0.92	1.86	0.06
Language_English	0.06	0.00–0.75	-2.84	0.86	-2.18	0.03
Language_French	4.82	0.79–29.26	1.57	0.92	1.71	0.09
Language_German	1.45	0.27–7.79	0.37	0.86	0.44	0.66
Multilingual_status	0.87	0.52–1.48	-0.13	0.27	-0.50	0.62

Lines in bold correspond to significant predictors (*p* < .05)

Additionally, a significant main effect of grammatical correctness was found (β = - 1.14, SE = 0.31, *p* < .001), with grammatically correct sentences yielding better performance than grammatically incorrect sentences, be they semantically odd or not. Similarly, a significant effect of semantic appropriateness was found (β=  -0.50, SE = 0.23, *p* < .05), with semantically appropriate sentences exhibiting better performance than sentences with anomalous meaning, irrespective of their grammatical status (Online Appendix E). The non-significant interactions between groups and the effect of grammatical correctness (β= - 0.76, SE = 0.50, *p* > .05) as well as between groups and the effect of semantic appropriateness (β=-0.56, SE = 0.31, *p* = .07) does not enable us to conclude whether groups were differently impacted by these effects. However, the interaction between the effect of grammatical correctness and semantic appropriateness was significant (β = 3.38, SE = 0.51, *p* < .001), suggesting that the effect of grammatical correctness varies depending on the semantic status of the sentence, and vice-versa. In other terms, this translates to a congruency effect, where sentences that are both grammatically correct and semantically appropriate (G + S+) or grammatically incorrect and semantically odd (G - S -) are performed better than incongruent sentences where the two linguistic dimensions are conflicting, i.e., sentences that are grammatically correct but semantically odd (G + S-) and sentences that are grammatically incorrect but semantically appropriate (G - S +). Controlling for all other demographic, cognitive and linguistic variables, the English-speaking participants displayed significantly lower performance than the overall performance of all language groups (β= - 2.84, SE = 1.31, *p* < .05). The English-speaking group indeed exhibited an overall mean performance of 76,7%, while the French-speaking group produced 84.2% of correct response, the German-speaking group 85.2% and the Italian-speaking 79.9%. Post-hoc investigations show that, as expected, sentences containing a syntactic error (i.e., determiner-name reversal) were generally better identified as ungrammatical, in comparison to sentences containing a subject-verb agreement mismatch. This effect was however particularly striking for the English-speaking group, which performed on average below chance level on sentences containing morphological disagreement but not on sentences with syntactic reversal (Online Appendix F).

## Discussion

This study explored the metalinguistic awareness skills of 6-to-12 year-old autistic children, by examining their performance in a low-verbal GJT engaging two dimensions of metalinguistic skills (i.e., *metasemantic* and *metamorphosyntactic awareness*) in comparison to age-matched neurotypical peers of comparable receptive morphosyntactic abilities, non-verbal reasoning skills, and socioeconomic status. A logistic mixed effects analysis was conducted to evaluate (1) whether variations in performance between groups were present and (2) whether the type of sentence differently affected their accuracy. Accounting for age, receptive morphosyntactic skills, non-verbal reasoning, and socio-economic status in addition to the language of administration, the model did not show a significant effect of group, neither on the traditional part of the task, engaging *metamorphosyntactic* skills, nor more surprisingly on the part involving an additional demand in *metasemantic* abilities (i.e., sentences with anomalous meaning). These results could suggest a comparable pattern of performance on a metacognitive task between children with ASD and their peers with TD, possibly thanks to being minimally linguistically and cognitively taxing, but it is important to remain cautious given that such an interpretation cannot be confirmed by the absence of significant difference between the groups. Nevertheless, in our task, autistic participants were able to mobilize metacognitive resources, and this ability to judge sentences based on grammatical correctness aligns with previous work conducted in older children with ASD and adolescents (Ambridge et al., 2015; Eigsti & Bennetto, 2009). This ability was primarily observed for shorter sentences, sentences with less subtle grammatical errors, such as word order manipulations, yes/no questions violations, and determiners substitutions and omissions (Eigsti & Bennetto, 2009). Our findings also show that autistic participants can access form as distinct from meaning.

Pertaining to semantic awareness, no difference in comparison to neurotypical peers could be demonstrated on sentences with anomalous meaning, which contradicts the existing literature showing difficulties for autistic individuals in understanding sentences with odd meaning, and dealing with figurative language in general (see Kalandadze et al., 2018; Lampri et al., 2023; Morsanyi et al., 2020). We argue that these seemingly contradictory findings may in fact be the result of methodological differences: as participants in our task were asked to judge the items based on their grammatical content only, and not on their semantic appropriateness, the current task arguably relies less on *metasemantic* resources compared to other tasks used in the previous studies. For instance, formulating two distinct interpretations to resolve the ambiguity in a sentence heard in Lewis et al. (2007)’ *ambiguous sentences* subtest might impose a high demand in linguistic skills to understand the sentence and justify one’s answer verbally, in addition to an effort in conceptualization and potentially cognitive flexibility to conceive two alternate, plausible solutions. Similarly, processing figurative language such as metaphors is known to require the coordination of complex linguistic and cognitive skills i.e., in (1) processing the actual literal meaning of the sentence, (2) detecting the ambiguity and (3) mapping it with the intended message. Instead, the current GJT mobilized *metamorphosyntactic* and *metasemantic* skills in having to distinguish form from meaning. In order to more equally flag both linguistic levels in future work, the task should include instructions to judge the sentences based on the meaning, irrespective of the sentences’ grammatical status, thus soliciting judgments on both the morphosyntactic and semantic dimensions alternately. Moreover, as our task implicated ignoring potentially misleading meanings in half of the trials, it would have been interesting to explore the extent to which the participants’ performance specifically in sentences that are grammatically correct but semantically anomalous is associated with their executive function abilities (i.e., set of higher-order cognitive skills enabling to achieve a goal such as e.g., cognitive flexibility, inhibitory control, working memory (Diamond, 2013), as shown in previous studies investigating multilingual effects on similar tasks targeting metalinguistic awareness (Bialystok & Ryan, 1985; Friesen & Bialystok, 2012). As executive functions have been shown to be impaired in the autistic population (Hill, 2004), exploring their association with metalinguistic awareness might shed light on the underlying mechanisms used by the groups to perform the task. Our findings of seemingly comparable performance on these sentences displayed by our participants with ASD in comparison to TD peers could be explained by relatively high executive functions exhibited by our sample, that could have compensated for difficulties in metacognition – an assumption that would need to be experimentally measured and tested to be ascertained. In addition, the formal, rule-based nature of the semantic anomaly involving the recognition of an animacy disagreement in most of the semantically odd sentences (i.e., as in “the *blanket* is *jumping*”), could have enhanced the autistic participants’ preserved accuracy in these sentences^3^. Future research is warranted to specifically investigate this possibility.

For both groups, sentence types were found to play a significant role on the performance: firstly, there was an effect of grammaticality, implying that grammatically correct sentences were better performed than ungrammatical sentences, irrespective of their meaning being appropriate or not. Secondly, an effect of semantic appropriateness was also identified, suggesting that semantically appropriate sentences were better performed than sentences with semantically odd meanings, be they grammatically correct or incorrect. Crucially, the interaction between these two effects resulted in a congruency effect, a well-known effect in psycholinguistic experiments: congruent sentences (i.e., sentences correct in both their grammatical and semantic dimensions (G + S+), or incorrect in both dimensions (G - S -) generated better scores than incongruent sentences (i.e., sentences containing conflicting dimensions where only one dimension is incorrect, as in G + S- and G - S + sentences). This lower performance on incongruent sentences likely highlights the complexity of this task imposed by the intertwinement of the *metasemantic* and *metamorphosyntactic* dimensions. This aligns with the positive main effect of non-verbal reasoning, which was identified as a significant predictor of performance: participants with higher IQ also had better performance in the task. Crucially, both underscore that participants did engage in the task and did not reply randomly, as the scores consistently exceeded chance level^4^. Consistent with previous studies ascertaining a gradual development of metalinguistic awareness (Edwards & Kirkpatrick, 1999; Melogno et al., 2022; Sinclair, 1986), the performance on the task significantly improved with age, after accounting for all other variables.

Despite our best efforts to make the GJT’s instructions, structure, and stimuli as similar as possible across all languages of administration, the English-speaking participants did exhibit lower performance in comparison to the other language groups. Beyond the plausible presence of individual differences not captured by the model which could lead to differences in performance, the lower performance of the English-speaking participants could be potentially linked to the experimental material (i.e., the audio stimuli): as compared to the other languages, the English native speaker who recorded the audio stimuli could have spoken faster, plausibly resulting in a greater difficulty to keep track of the information in the sentences and, more specifically, to detect the grammatical errors that were short in duration (i.e., morphological verb-agreement mismatch and syntactic noun-determiner reversal). A more convincing explanation pertains to the lack of detection of the morphosyntactic mistakes in the grammatically incorrect sentences by the English-speaking participants: specifically, post-hoc investigations revealed that English participants did not identify sentences with morphological disagreement above chance level. This can be due to the fact that the -s agreement marker shows variation in its use across different varieties of English where it can be absent (Patrick, 2008; Sedlatschek, 2009; Wolfram & Schilling, 2015), and, when present, is likely to be omitted in language impairment (Brown, 1973; Rice & Wexler, 1996)^5^. This limitation could have only a minimal repercussion on the current findings, and therefore does not cast doubt on their robustness, as the language version was controlled for in being entered as a covariate in the statistical model. It nevertheless underscores the challenges of testing in multiple languages (Hambleton & Patsula, 1998; Iliescu, 2017), although this is essential to promote scientific collaborations to achieve larger cohorts and consequently to gain more statistical power *(see* Prévost & Tuller, 2022*for an in-depth discussion of this endeavor in studies on autism)*.

The study’s main limitation might stem from the sample constituting the ASD groups: firstly, there were only 38 participants included in the autistic group, as participants had to meet sufficient criteria in terms of linguistic and cognitive (e.g., attentional) resources to complete the entire assessment (GJT, non-verbal IQ test, receptive morphosyntactic assessment). Consequently, only a few participants in our sample presented an intellectual impairment, resulting in a possible selection bias, a common issue ubiquitous in autism research (Russell et al., 2019). Secondly, most of these children were recruited from higher socioeconomic backgrounds, constituting a WEIRD (Western, Educated, Industrialized, Rich and Democratic) sample, limiting its generalizability across diverse cultures and contexts (Henrich et al., 2010). Altogether, these points also raise the question about the representativeness of our sample with respect to the heterogeneity of the linguistic, social, and cognitive profiles in autistic individuals.

Nevertheless, this work offers interesting perspectives in both the educational and research fields: the instruction of morphosyntactic awareness has demonstrated positive effects on literacy development and vocabulary knowledge (Goodwin & Ahn, 2010; Ramirez et al., 2014), even benefiting struggling learners (Deacon et al., 2008) in the neurotypical population. By extending these strategies to children with ASD, who seem to possess a baseline level of morphosyntactic awareness, our study suggests potential advantages for the ASD population. As multilingual effects on metalinguistic awareness have been reliably found in the neurotypical population (Adesope et al., 2010) with bilinguals outperforming monolinguals in a range of tasks of *metamorphosyntactic* and *metasemantic* nature (Ben-Zeev, 1977; Bialystok, 2001; Hermanto et al., 2012), investigating these effects in the autistic population could provide valuable insights into the underlying linguistic and cognitive strategies at play in this growing but understudied population (Beauchamp & MacLeod, 2017; Prévost & Tuller, 2022). Proposing multilingual tools such as the GJT developed for the sake of this study might be particularly suitable to achieve higher cohorts and enable testing in different regions children with various linguistic backgrounds.

To our knowledge, this study is the first to explore *metamorphosyntactic* and *metasemantic* competence of school-aged autistic children in comparison to aged-matched neurotypical peers of comparable non-verbal reasoning and receptive linguistic skills, in a gamified task of reduced linguistic complexity that does not require a verbal response. The current findings seem to suggest that autistic children may be as able as their neurotypical peers to mobilize metalinguistic skills at the morphosyntactic and semantic levels in a task of reduced linguistic complexity.

## Electronic supplementary material

Below is the link to the electronic supplementary material.

Supplementary Material 1

## Data Availability

Data and code are available on OSF repository, under: https://osf.io/wnsdc/?view_only=4baca7db03d24759b4d695b59990c112.

## Footnotes

1 Both person-first and identity-first language will be used indistinctively throughout the manuscript to refer to individuals with an Autism diagnosis, so as to reflect the variety of preferences amongst the autistic community (Bottema-Beutel et al., 2021).

2 We thank an anonymous reviewer for drawing this to our attention.

3 We thank an anonymous reviewer for evoking this possibility.

4 A reviewer raised the possibility that some children could have adopted a candy-heavy approach (i.e., systematically selecting the candy instead of the sock to please the monster). Post-hoc analyses revealed that only 3 participants (2 in the group of children with ASD, 1 in the group of children with TD) might have adopted this approach.

5 We thank the reviewers for their expertise in suggesting this explanation.
