# Supplementary material for: Exploring Metalinguistic Awareness in School-Aged Autistic Children: Insights from Grammatical Judgment
Source: J Autism Dev Disord. 2024 Oct 7;56(2):560–73. doi: 10.1007/s10803-024-06569-y (PMC12864310; doi:10.1007/s10803-024-06569-y)

**Supplementary Material – Online Appendix**

**Appendix A**. Languages multilingual children were exposed to (more than >20% of their lifetime), in addition to the language in which they were tested.

| **Language of test** | **English**  **N=3** | **French**  **N=16** | **German**  **N=23** | **Italian**  **N=4** |
| --- | --- | --- | --- | --- |
| **Additional languages multilingual participants were exposed to…** | German (N=3) | Arabic (N=1) | Chinese (N=1) | Dutch (N=1) |
|  |  | English (N=4) | Czech (N=1) | German (N=1) |
|  |  | German (N=4) | Dutch (N=1) | Macedonian (N=1) |
|  |  | Hungarian (N=1) | English (N=2) | Serbo-Croatian (N=1) |
|  |  | Italian (N=1) | Italian (N=4) |  |
|  |  | Japanese (N=2) | Portuguese (N=1) |  |
|  |  | Malagasy (N=1) | Russian (N=1) |  |
|  |  | Spanish (N=1) | Spanish (N=11) |  |
|  |  | Swiss-German (N=1) | Swiss-German (N=1) |  |

**Appendix B**. Item list in all languages. Blanks refer to initial stimuli that have been removed after the pilot phase with neurotypical adults, so as to keep 4*8 items per language. Original stimuli names have been kept for transparency reasons, to be able to better compare the languages. The item “gramm_sem_8” has been removed from all analyses after a reviewer’s suggestion (see footnote 2).

| **Item** | **French** | **English** | **German** | **Italian** |
| --- | --- | --- | --- | --- |
| practice | Tu bois du thé. | You drink tea. | Du trinkst Tee. | Tu bevi il tè |
| practice | La dame dort. | The woman sleeps. | Die Dame schläft. | La signora sta dormendo |
| practice | Elle fromage mange. | She cheese eats. | Sie Käse isst. | Lei formaggio mangia |
| practice | Il pain coupe. | He bread cuts. | Er Brot schneidet. | Lui pane taglia |
| practice | La balle chante. | The ball is singing. | Der Ball singt. | La palla canta |
| practice | La table marche. | The table is walking. | Der Tisch läuft. | ll tavolo cammina |
| **gramm_sem_1** | Le grand-père jette le mouchoir à la poubelle. |  | Der Großvater wirft das Tuch in den Mülleimer. |  |
| **gramm_sem_2** | Le bébé fait la sieste dans le lit. | The baby is taking a nap in a bed. | Das Baby schläft heute im Schlafzimmer. | Il bambino dorme sul’ letto |
| **gramm_sem_3** | La fille écrit des histoires dans un livre. | The girl is writing stories in a book. | Das Mädchen schreibt Geschichten in ein Buch. | La donna scrive storie in un libro |
| **gramm_sem_4** | Tu laves le pyjama dans la machine. | You wash the pyjamas in the machine. | Du wäschst das Hemd in der Waschmaschine. | Tu lavi la giacca in lavatrice |
| **gramm_sem_5** |  |  | Im Wald gibt es Hasen und Füchse. | C'è un coniglio nel giardino |
| **gramm_sem_6** | La grand-mère lit un livre sur un banc. | Grandmother is reading a book on a bench. | Die Großmutter liest ein Buch auf einer Bank. | La ragazza legge un libro |
| **gramm_sem_7** | Le docteur fait un sourire au garçon. | The doctor is smiling at the boy. |  | Il dottore saluta il ragazzo. |
| **gramm_sem_8** |  | **~~The lady raises the hand to say Hello~~.** |  |  |
| **gramm_sem_9** | Marc va à la piscine avec un ami. | Mark goes to the swimming pool with a friend. | Mark geht mit einem Freund ins Schwimmbad. | Marco va al mare con un amico. |
| **gramm_sem_10** | J’allume la lumière dans le couloir. | I am turning the light on in the hallway. | Ich schalte das Licht im Wohnzimmer ein. | Accendo la luce nella casa |
| agramm_sem_1  *(agreement)* | Tu préparont une soupe pour le diner. | The man open the door of the house. | Der Herr kochen eine Suppe am Abend. | Tu cucinate la carne la sera. |
| agramm_sem_2  *(agreement*) |  | The hen lay eggs every morning. |  | Le scimmie mangia delle banane |
| agramm_sem_3  *(agreement)* | Le poisson et le canard nageons dans l’eau. | The duck are swimming in the water. | Die Ente und der Fisch schwimmt im Wasser. | La rana nuotiamo nell'acqua |
| agramm_sem_4  *(agreement)* | L’enfant achetons des pommes et des bananes. | The children buys bananas. | Das Kind kaufen Eis im Supermarkt. | Il bambino comprano frutta |
| agramm_sem_5  *(agreement)* | Le pompier conduire le camion sur la route. |  | Der Feuerwehrmann fährst mit dem Lastwagen. |  |
| agramm_sem_6  *(reversal)* | Tu nettoies la baignoire dans salle la de bains. |  | Mädchen das spielt vor dem Haus mit dem Ball. | Ragazza la gioca a palla |
| agramm_sem_7  *(reversal)* |  | You are cleaning bathtub the in the bathroom. |  | Maria pulisce cucina la |
| agramm_sem_8  *(reversal)* | Marie cueille des fleurs dans jardin le. | Mary is picking flowers in garden the. | Theresa fährt mit Zug dem in die Stadt. |  |
| agramm_sem_9  *(reversal)* | Elle porte une robe et chaussures des. | She is wearing scarf a and a dress. | Sie trägt Strumpfhose eine und Kleid ein. | Porta una gonna e scarpe delle |
| agramm_sem_10  *(reversal)* | La nuit il y a nuages des dans le ciel. | Tonight there are clouds and stars in sky the. | Es gibt in Nacht der Wolken am Himmel. | Di notte sono stelle ci in cielo. |
| **gramm_asem_1** | Le balai se réveille tous les matins. | The broom wakes up every morning. | Der Besen wacht jeden Morgen auf. | La scopa si sveglia la mattina |
| **gramm_asem_2** |  |  |  |  |
| **gramm_asem_3** | La chaussure cuisine des spaghettis. | The shoe is cooking spaghetti and eggs. | Der Schuh kocht Spaghetti mit Käse. | La scarpa cucina gli spaghetti |
| **gramm_asem_4** | La brosse à dent est fatiguée ce soir. | The toothbrush is tired and wants to sleep. | Die Zahnbürste ist heute Abend müde. | Il telefono è stanco stasera |
| **gramm_asem_5** | Les fourchettes et les couteaux jouent dans le parc. | Forks and knives are playing in the park. | Die Gabeln und Messer spielen im Park. | Piatti e tazze giocano nel mare. |
| **gramm_asem_6** | Le magasin chante à la radio. | The shop is singing on the radio tonight. | Der Staubsauger singt im Radio. | Il negozio canta alla radio |
| **gramm_asem_7** | La bouteille se dépêche et court dehors. | The bottle hurries and runs in the bedroom. | Die Flasche beeilt sich und läuft nach draussen. | La scopa si sbriga e corre fuori |
| **gramm_asem_8** |  |  |  |  |
| **gramm_asem_9** | Aujourd’hui le frigo coupe un gâteau. | Today the fridge is cutting a cake. | Der Kühlschrank schneidet heute eine Torte. | Oggi il forno taglia una torta |
| **gramm_asem_10** | Les compotes de fruits écoutent la maitresse. | The kitchen listens to the teacher. | Die Küche hört der Lehrerin zu. | La cucina ascolta la pediatra |
| agramm_asem_1  *(agreement)* |  |  | Die Garage schüttelst die Suppe. | Il bar baciare la caramella |
| agramm_asem_2  *(agreement)* | L'éléphant dormons avec le bateau. | The elephant sleep with the boat. | Der Elefant schlafen mit dem Boot. | L'elefante dormono con la barca |
| agramm_asem_3  *(agreement)* | La piscine recevoir un savon au cirque. | The pool is get a soap at the circus. | Der Teich kriegen eine Seife im Zirkus. | La doccia dare un libro al circo. |
| agramm_asem_4  *(agreement)* | La pluie grimpons ce soir dans le caillou. | The rain climb tonight in the sandbox. | Der Regen klettern heute Nacht in den Stein. | La pioggia si arrampicare sul sasso |
| agramm_asem_5  *(agreement)* | Les lunettes donnons un genou à la plage. | The glasses gives a knee at the beach. |  |  |
| agramm_asem_6  *(reversal)* | Le panier attend train le à l'école. | Basket the is waiting for the train at school. | Der Korb wartet auf Zug den in der Schule. | La scopa aspetta treno il |
| agramm_asem_7  *(reversal)* | La couverture saute sur pommes de les terre. | The blanket is jumping on potato the. | Die Decke springt auf Kartoffeln die. | La coperta salta patate sulle |
| agramm_asem_8  *(reversal)* | Le facteur glisse fleur la dans le ciel. | The mailman slides flowers in sky the. | Der Briefträger rutscht Blumen in Himmel den. | La parrucchiere si siede cielo nel |
| agramm_asem_9  *(reversal)* |  |  |  |  |
| agramm_asem_10  *(reversal)* | La veste attend biberon le dans l'escalier. | The jacket waits for the box in staircase the. | Die Jacke wartet auf Flasche die. | La giacca aspetta bottiglia la |

**Appendix C. Additional information on the statistical approach, including contrast coding, model specifications, checks and assessment.**

*Variable scaling*. All continuous variables were centered around the mean.

*Missing data*. There was no missing data in the variables age, non-verbal IQ, receptive morphosyntactic score, socioeconomic status, language, and GJT scores: only the participants who completed these tasks entirely were included.

*Model checks*. Prior to conducting the model, we ensured that there was no potential harmful collinearity between the variables: all variance inflation factors were below 5.

*Contrast coding.* The group variable was recoded using a scaled sum contrast: children with TD were assigned a value of -0.5, while the group of children with ASD was assigned a value of 0.5; thus, a negative estimate for this variable suggests that the autistic group exhibited lower performance. For the variable *effect of grammaticality*, grammatically correct sentences were assigned a value of -0.5, and 0.5 to grammatically incorrect sentences. Similarly, a value of -0.5 was allocated to sentences with appropriate meaning, while 0.5 was assigned to sentences with odd meaning, for the variable *semantic appropriateness*. In this way, positive estimates for these variables reflect better performance for grammatically correct and semantically appropriate sentences, respectively. Finally, a scaled sum contrast was set to recode the variable *language of administration,* reflecting the language in which the child performed the task to account for different language versions; it allowed for the comparison of each language group against the overall mean of performance in all languages.

*Model selection*. A first model containing the variables mentioned (i.e., biological age (in months), non-verbal IQ (Ravens’ score), general receptive morphosyntactic skills (TROG z-score), socioeconomic status proxy (Likert scale score 1-5), language of administration (English, French, German or Italian) as well as bilingual status (monolingual/bilingual)), as well as a 3-way interaction term between group, grammatical correctness and semantic appropriateness was conducted. The 3-way interaction term was not significant and was therefore removed. The model with two ways interactions did not converge and was simplified by removing the correlations between random intercepts and random slopes, yielding the final model used.

*Model assessment*. The model’s total explanatory power was substantial (conditional R2 = 0.55), and its part related to the fixed effects alone (marginal R2) accounted for 0.34.

**Appendix D**. Groups’ performance on the four sentence types. G+/G- hold for grammatically correct or incorrect, S+/S- for semantically appropriate or odd, respectively.


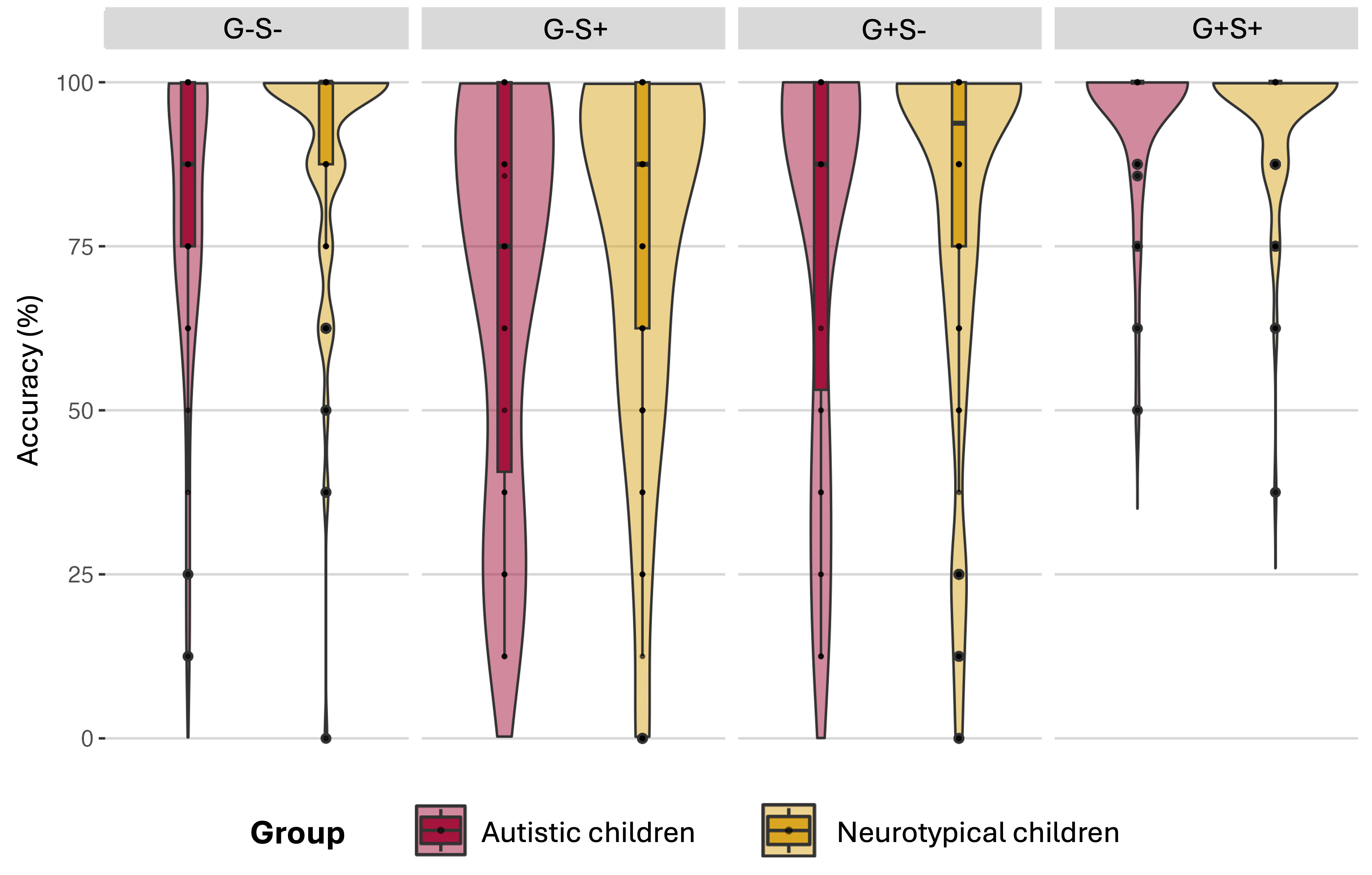


**Appendix E.** Percentage of errors per groups depending on semantic appropriateness and grammatical correctness. G+/G- hold for grammatically correct or incorrect, S+/S- for semantically appropriate or odd, respectively

|  | **G +** | **G -** | **S +** | **S -** |
| --- | --- | --- | --- | --- |
| Children with TD | 12.4% | 18.1% | 15.4% | 15.2% |
| Children with ASD | 13.8% | 24.7% | 17.6% | 20.8% |
| All children | 12.8% | 20.1% | 16.0% | 16.9% |

**Appendix F.** Language groups’ performance on grammatically incorrect sentences (N=8 semantically appropriate and N=8 semantically odd), as a function of the type of error it contains (reversal or agreement).


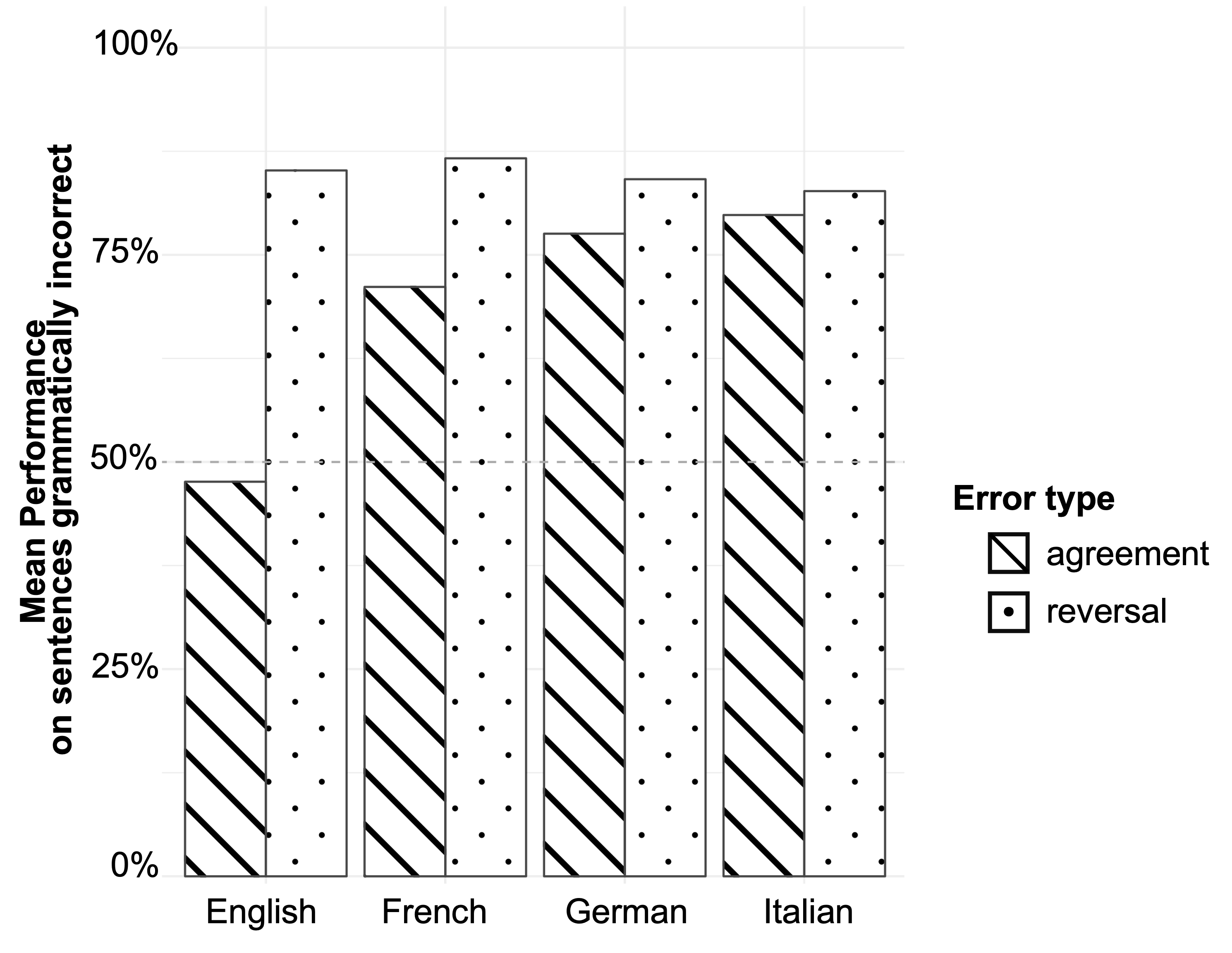

Supplement: Supplementary file 1 — Supplementary Material 1 [file 10803_2024_6569_MOESM1_ESM.docx]
